# Conference Report: FORTY-SEVENTH ANNUAL GASEOUS ELECTRONICS CONFERENCE Gaithersburg, MD October 18–21, 1994

**DOI:** 10.6028/jres.100.037

**Published:** 1995

**Authors:** Richard J. Van Brunt, Jean W. Gallagher

**Affiliations:** Electricity Division, National Institute of Standards and Technology, Gaithersburg, MD 20899-0001; Technology Services, National Institute of Standards and Technology, Gaithersburg, MD 20899-0001

## 1. Introduction

The Gaseous Electronics Conference (GEC) is an annual topical conference of The American Physical Society (APS) sponsored by the APS Division of Atomic, Molecular, and Optical Physics. The traditional focus of this conference is on basic processes and phenomena in relatively low-temperature plasmas and weakly ionized gases such as encountered in planetary atmospheres, lighting systems, lasers, plasma processing reactors, and electrical breakdown and discharges in gases. Particular attention is given to relatively low-energy ionization and collision phenomena needed to understand and model gas discharges such as electron-molecule collisions, ion-molecule reactions, electron-ion and ion-ion recombination, photoionization, photodetachment of negative ions, ion-cluster formation, and ion-, photon-, electron-, and metastable-surface interactions. Among the topics covered by the GEC are: 1) plasma diagnostics and measurement methods, 2) theory and simulation of plasmas, 3) plasma chemistry, 4) ion and electron transport in gases, and 5) etching, sputtering, and deposition at surfaces. In recent years, the GEC has become an important international forum for discussing research on or relevant to the fundamental aspects of plasma reactors such as used for plasma processing of materials.

The 47th GEC was hosted by and held at the National Institute of Standards and Technology (NIST) in Gaithersburg, Maryland on October 18–21, 1994. Special topics highlighted at this conference included: electron-metal atom collisions, recent developments in electron-molecule scattering, negative ions in plasmas, plasma displays, diamond-film deposition, plasma treatment of waste gases, and innovative plasma applications. The Will Allis Prize Lecture was delivered by Eldon E. Ferguson of the National Oceanographic and Atmospheric Administration (NOAA) in Boulder, Colorado.

In conjunction with the 1994 GEC, there was a meeting of the GEC Reference Cell Users Group. The “GEC RF Reference Reactor,” presently in use at NIST and in many other laboratories around the world, was the outgrowth of a special session held at the 1988 GEC in Minneapolis, Minnesota. The present special issue of the NIST Journal of Research is devoted to recent work performed in different laboratories that is directly related to the GEC reference cell.

## 2. Conference Organization

The conference program included over 300 papers presented in either two parallel oral sessions or in combined poster sessions. Thirty-six oral and poster sessions were organized according to particular topics that included: high-density plasma processing, electron-metal atom collisions, negative ions in plasmas, plasma diagnostics and control, plasma-surface interactions, magnetically enhanced plasmas, charged particle transport and energy distributions, inductively coupled plasmas, electron-molecule scattering, diamond films, glows, plasma techniques for waste treatment, innovative plasma applications, dusty plasmas, microwave discharges, plasma chemistry, heavy particle interactions, cathodes and sheaths, rf capacitively coupled glows, electron-ion recombination and photon processes, and plasma etching and deposition.

The 1994 GEC Chairperson was James T. Dakin of GE Lighting. Richard J. Van Brunt of the NIST Electronics and Electrical Engineering Laboratory (EEEL) served as the GEC Secretary and Jean W. Gallagher of NIST Technology Services served as a member of the GEC Executive Committee. James K. Olthoff of NIST-EEEL chaired the meeting of the GEC Reference Cell Users Group held in conjunction with the GEC and also served on the local organizing committee. Katharine Gebbie, Director of the NIST Physics Laboratory, and Thomas Weber of the National Science Foundation were the banquet speakers. The National Institute of Standards and Technology, formerly the National Bureau of Standards (NBS), has traditionally been a major contributor to and sponsor of the GEC over the years. At the 1994 GEC, there were 14 papers presented by NIST authors from the EEEL, Physics Laboratory, and the Chemical Science and Technology Laboratory both from Gaithersburg and from Boulder. The 12th GEC (1959) was hosted by NBS in Washington, DC and the 21st (1968) and 37th (1984) were hosted by NBS in Boulder, Colorado. It is interesting to note that 51 papers were presented at the 1959 conference compared to 302 at the 1994 conference. Although the GEC has grown in size, the number of contributions at the four most recent conferences has leveled off at about 300. It appears that some of the growth in the past decade can be attributed to an increase in foreign participation. At the 1994 GEC there were delegates from numerous foreign countries including Canada, United Kingdom, Germany, France, Japan, Russia, Portugal, Italy, Ireland, The Netherlands, Australia, Mexico, India, Brazil, Israel, Poland, and Serbia.

Abstracts for most of the papers presented at the 1994 GEC have been published in the Bulletin of the American Physical Society [1]. There were approximately 340 attendees at the conference. A list of the attendees is available from the Conference Secretary who can be contacted by electronic mail (vanbrunt@eeel.nist.gov) or by fax (301-948-5796). The 1995 GEC will be hosted by the University of California at Berkeley. Information about this conference can be obtained from the 1995 Conference Secretary:

David Graves
Department of Chemical Engineering,
University of California,
Berkeley, CA 94720, USA
Telephone: 510-642-2214
Fax: 510-642-4778
Email: graves@condor.cchem.berkeley.edu.

The GEC has always taken place in North America. In view of the increasing international participation, it was suggested that some future conferences should be held outside of North America. A proposal was submitted by Ian Falconer from the University of Sydney to hold the GEC in Australia in about 4 years. This proposal is now under consideration by the GEC Executive Committee.

## 3. Technical Highlights

Due to the large number of papers presented at the GEC, it is possible to discuss only a small fraction of them in this report. Selected highlights from some of the presentations are given here to provide a flavor of the scope and content of the conference. More complete information about the scientific and technical content can be found in the published abstracts [1].

A timely invited lecture demonstration of a revolutionary new microwave light source was presented by Michael Ury and his coworkers from Fusion Lighting, Inc., Rockville, Maryland. The day before the GEC lecture, the first large-scale test installation of the light source was unveiled at Department of Energy’s Forrestal Building headquarters in Washington, DC [2]. Another test is being conducted using a similar system installed at the National Air and Space Museum in Washington. The light source is based on an electrodeless microwave discharge in sulfur vapor contained in a long tube, and produces efficient, continuous, visible radiation with high color rendering characteristics. In the test at the Air and Space Museum, “three 90 foot light pipes powered by sulfur bulbs have replaced 94 separate conventional fixtures in one display area.” The theory and application of the new light source were discussed in the lecture during which the operation of a prototype light pipe was demonstrated.

Another timely invited lecture was presented by Yuri Raizer from the Institute for Problems in Mechanics, Moscow, Russia. Professor Raizer is the recipient of the most recent F. M. Penning Award at the International Conference on Phenomena in Ionized Gases and is an internationally recognized expert on the theory of electrical discharges. His invited lecture concerned the basic theory of dc and rf glow discharges. It roughly coincided with the release of his most recent book published by CRC Press on the theory of capacitively coupled rf discharges [3].

Loucas G. Christophorou, formerly from the Oak Ridge National Laboratory (ORNL) and now at NIST, chaired an interesting session on the role of negative ions in plasmas. Frederick J. de Hoog of the Eindhoven University of Technology in the The Netherlands presented an invited talk in this session about the important and difficult problems of measuring or observing negative ions in plasmas. For electronegative gases like O_2_, SF_6_ and CF_4_, negative ions play an important role in the discharge physics as was pointed out in a presentation by Toshiaki Makabe and coworkers from Keio University, Japan, on modeling of O_2_ rf glow discharges. Measurement of negative ions in rf discharges remains a difficult problem. Brian Smith and Larry Overzet from the University of Texas at Dallas reported on the first measurements of negative ion kinetic energies in an etching plasma. Panos Datskos and Loucas Christophorou of ORNL reported important new results on photodetachment of the negative ions ${\mathrm{SF}}_{6}^{-}$ and $C_{6}F_{6}^{-}$. In the past, there has been considerable uncertainty and controversy about the photodetachment threshold for ${\mathrm{SF}}_{6}^{-}$ which this work and a recently published work by a German group [4] have helped to resolve.

Three papers were presented from different laboratories on plasma chemistry in SF_6_ and SF_6_/O_2_ corona and spark discharges: one from France, one from Canada, and one that resulted from a collaborative effort involving the Hydro Quebec Research Laboratory, ORNL, and NIST. One of the important issues addressed by these papers was the influence of electrode material, oxygen, and water vapor on the formation of the highly toxic compounds S_2_F_10_ and S_2_O_2_F_10_. The results reported confirm previous work which showed that S_2_F_10_ is formed by decomposition of SF_6_ in discharges, and show additionally that the rate of S_2_F_10_ formation is dramatically reduced when small quantities of O_2_ are introduced in SF_6_ (up to 10 % by gas volume ratio). The presence of O_2_, however, was found to significantly enhance S_2_O_2_F_10_ production in a corona discharge and S_2_OF_10_ production in a spark. The results can be understood using a plasma chemical model recently developed at NIST [5] and are relevant to the use of SF_6_ as a gaseous dielectric in electrical power systems.

Numerous papers were presented about recent investigations of electron-atom and -molecule processes of obvious importance in electrical discharges. Donald H. Madison of the University of Missouri-Rolla gave an invited talk about the status of total cross sections for electron scattering from atomic and molecular hydrogen that are particularly relevant to understanding plasmas used to prepare diamond films. Bernhard Stumpf from the University of Idaho gave an invited talk about recent experimental work on electron-metal atom scattering, and Klaus Bartschat from Drake University reviewed the recent theoretical work on the same subject. Considered in these talks were various metal atoms ranging from relatively simple heavy alkali targets to complex species such as copper, silver, iron, mercury, and lead. Yong-Ki Kim of NIST chaired the session on electron-metal atom collisions and also presented a paper about his own work on a simple, effective model for generating electron-impact ionization cross sections for atoms and molecules which was developed in a collaboration with M. E. Rudd of the University of Nebraska. This model, which they call the Binary-Encounter-Dipole (BED) model, reproduces known ionization cross sections for species such as H, He, He^+^, Li^+ +^, Ne, and H_2_ to within 10 % from threshold to a few keV in incident electron energy. Papers were presented on many aspects of electron-molecule collisions including electron collisions with metastable excited species; electron-ion recombination of molecular ions such as O_2_H^+^, N_2_OH^+^, ${\mathrm{CS}}_{2}^{+}$, HCO^+^, N_2_H^+^, and $H_{3}^{+}$; electron excitation of numerous molecular species such as C_2_F_6_, C_3_F_8_, SF_6_, CF_4_, N_2_, NO, and NO_2_; electron-impact ionization of species such as SO_2_ and tetramethylsilane; and electron attachment to ground-state and optically-excited molecules.

There were 17 talks and posters (nearly 6 % of the total contributions) concerned with the GEC rf-discharge reference cell at the 1994 conference. These presentations covered many different investigations that utilized or directly pertained to the reference cell. Among the diverse topics relevant to the reference cell were: 1) argon metastable density measurements by laser-induced fluorescence, 2) electrical impedance characteristics of the rf plasma sheaths and glow regions, 3) comparison of microwave interferometer and Langmuir probe determinations of electron density, 4) ion energy distributions in H_2_, N_2_, O_2_, He and Ar/N_2_ discharges, 5) temporally and spatially resolved measurements of emission from the rf glow in H_2_, 6) the kinship of the reference cell to ac-plasma display cells, 7) rf discharge simulations applied to the reference cell, 8) formation of a Coulomb solid in dusty plasmas using the reference cell, 9) hydrogen atom density measurements in H_2_/N_2_ gas mixtures, and 10) adaptations of the reference cell to investigate inductively excited plasmas. Some of these topics are covered in the papers on the GEC reference cell that are published in the present special issue.

## 4. William P. Allis Prize Lecture

The William P. Allis Prize is awarded by the APS every 2 years for outstanding contributions in the field of gaseous electronics and is named in honor of Will Allis, Professor Emeritus at the Massachusetts Institute of Technology, who is one of the founders of the GEC and served as its Honorary Chairman until 1989. The recipient of this prize traditionally delivers a lecture at a plenary session of the GEC. At the 1994 GEC, the third Will Allis Prize Lecture was delivered by Eldon E. Ferguson, Director of the Climate Monitoring and Diagnostic Laboratory of NOAA in Boulder, CO. Eldon Ferguson talked about the development and early application of the flowing afterglow technique to investigate low-energy ion-molecule interactions. He was primarily responsible for this technique which he introduced in the early 1960s while employed by the National Bureau of Standards in Boulder. The present model of the earth’s ionosphere is largely based on the rates for ion processes that he and his colleagues measured at NBS and subsequently at NOAA. Eldon Ferguson has continued to maintain an affiliation with NIST by serving as a Fellow of the Joint Institute for Laboratory Astrophysics (JILA).

It is worth noting that Arthur V. Phelps of NIST-JILA received the first Will Allis Prize at the 43rd GEC (1990) held at the University of Illinois, Urbana-Champaign. Phelps, now retired from NIST, remains active in the field and presented two papers at the 1994 GEC describing results of his research on cathode-dominated, low-current and/or low-pressure discharges in argon and hydrogen.

## 5. Peripheral Meetings and Activities

A well attended meeting of the GEC Reference Cell Users Group was held in conjunction with the 1994 GEC. James K. Olthoff of NIST presided. The GEC rf discharge reference cell was the product of a special workshop held during the 1988 GEC in Minneapolis, Minnesota. Phil Hargis, Ken Greenberg, and Paul Miller of the Sandia National Laboratory took the lead in preparing the initial cell design. Subsequent input to the final design was received from a number of different laboratories including NIST. Implementation of the reference cell at NIST involved a cooperative effort among three different laboratories, namely the Physics, Chemical Science and Technology, and Electronics and Electrical Engineering Laboratories. The results from the initial tests and intercomparisons made using seven different cells set up at Sandia, NIST, the University of New Mexico, and the Wright Laboratory, Wright Patterson Air Force Base were published in 1994 [6]. Reference cells have now been installed in numerous other laboratories including the University of Michigan, Michigan Technological University, the University of Illinois, the University of Iowa, the Rochester Institute of Technology, Air Products, the Queens University of Belfast, and the University of Texas at Dallas. Efforts to model rf discharge behavior of the reference cell are underway at the University of Illinois, Scientific Research Associates, Auburn University, and Universite Paul Sabatier. Detailed information about the GEC reference cell concept and design are covered in the paper by Olthoff and Greenberg that appears in this issue [7].

During the User’s Group meeting, John Goree from the University of Iowa gave a presentation that included a short video about dusty plasma experiments performed using the GEC reference cell without an upper electrode. There was also a lively discussion about the types of experiments that can be performed with the reference cell and the extent to which the cell can be modified and still qualify as a “reference.” Plans for the preparation of this special issue of the NIST Journal of Research devoted to the reference cell were finalized at the meeting.

In conjunction with the 1994 GEC, meetings were also held of the National Research Council (NRC) Committee on Data Bases for Plasma Processing. These meetings were the prelude to a Workshop on Modeling, Simulation, and Database Needs in Plasma Processing that was held April 1–2, 1995, at the National Academy of Sciences in Washington, DC. The purpose of the workshop was to bring together experts first to assess the current status of plasma modeling and simulation, then to establish a prioritized list of database and diagnostic needs on the potential impact on integrated circuit manufacturing technology. The results of the workshop will be presented in an NRC report, which the Advanced Research Projects Agency (ARPA) has asked for to provide guidance in establishing new program emphases.

At the conclusion of the conference, technical tours of nine NIST laboratories were organized for the participants. Among the laboratories on the tour were those concerned with the vacuum standards, gaseous electronics, laser cooling of atoms, video standards, flat panel displays, the GEC reference cell, laser diagnostics and measurements in discharges, and nanolithography.
